# Associations Between the Built Environment in GPS-Derived Activity Spaces and Sedentary Behavior, Light Physical Activity, and Moderate-to-Vigorous Physical Activity

**DOI:** 10.3390/ijerph22040566

**Published:** 2025-04-04

**Authors:** Dante G. Vittor, Jeffrey S. Wilson, Scott E. Crouter, Benjamin G. Ethier, Ling Shi, Sarah M. Camhi, Philip J. Troped

**Affiliations:** 1 Manning College of Nursing and Health Sciences, University of Massachusetts Boston, Boston, MA 02125, USA; dantevittor@gmail.com (D.G.V.); ben.g.ethier@gmail.com (B.G.E.); ling.shi@umb.edu (L.S.); 2 Department of Geography, Indiana University Indianapolis, Indianapolis, IN 46202, USA; jeswilso@iu.edu; 3 Department of Kinesiology, Recreation, and Sport Studies, University of Tennessee Knoxville, Knoxville, TN 37996, USA; scrouter@utk.edu; 4 College of Arts and Sciences, University of San Francisco, San Francisco CA 94117, USA; scamhi2@usfca.edu

**Keywords:** accelerometry, built environment, environment, motion sensors, physical activity, sedentary behavior, activity space

## Abstract

Built environment and physical activity (PA) studies have predominantly used fixed or home-centric approaches to identify environmental exposures. In this study, GPS-derived daily activity spaces were used to examine the relationships between the built environment and sedentary behavior (SB), light PA (LPA), and moderate-to-vigorous PA (MVPA). Thirty-one adults were assessed with activity monitors and GPS units. Three types of activity spaces were created: 50 m buffered GPS tracks, minimum convex hulls (MCHs), and standard deviational ellipses (SDEs). The environmental variables included land use mix, greenness, and intersection, multi-use trail, bike infrastructure, and bike station densities. Repeated measures regression was used to test the associations for 141 person-days, controlling for age, gender, income, body mass index, crime, precipitation, and temperature. Greenness within MCH activity spaces was positively associated with LPA (*p* = 0.02). The bike infrastructure density within SDE spaces had a significant positive association with MVPA (*p* = 0.04). Multi-use trail, bike infrastructure, and bike station densities had significant negative associations with LPA (*p* ≤ 0.05). There were no significant adjusted associations with SB. The few significant associations in this study varied by outcome and type of activity space. Further studies are needed to determine optimal, yet flexible methods for activity spaces in built environment and PA research.

## 1. Introduction

Regular physical activity (PA) is an important contributor to health and wellbeing, including a decreased risk of chronic diseases and conditions, such as cardiovascular diseases, type 2 diabetes, and certain cancers [1]. These beneficial relationships were well established in the U.S. Surgeon General’s Report on Physical Activity and Health published in the mid-1990s [2] and in subsequent comprehensive reports on PA’s benefits published in 2008 and 2018 [1,3]. The global burden of physical inactivity on non-communicable disease morbidity and mortality has also been well documented in recent research [4]. Surveillance data indicate that PA trends are moving in a favorable but less-than-optimal direction. For example, one report based on data from the U.S. National Health Interview survey indicated that the prevalence of physical inactivity decreased from 40.5% in 1998 to 25.6% in 2018 and the prevalence of engaging in >300 min of moderate-intensity PA increased from 26.0% to 37.4% [5]. Globally, across 168 countries, the levels of insufficient PA, defined as less than 150 min of moderate-intensity PA or 75 min of vigorous-intensity or an equivalent combination of both, improved slightly from 28.5% in 2001 to 27.5% in 2016 [6].

To promote PA more effectively on a population level, a better understanding of the key influences on these behaviors is needed. The social ecological model is a framework that posits that the physical and social context to which people are exposed can help determine individual health behaviors, such as PA [7,8]. Increasingly, public health researchers and practitioners have focused on how the built or person-made environment influences PA. A recent review of studies in Europe indicated that a number of aspects of the built environment, such as the residential density, intersection density, parks, and transit stops, are positively related to objective measures of moderate-to-vigorous PA (MVPA) and self-reported walking for transport in adults [9]. Similarly a review of over 40 studies in older adults demonstrated consistent evidence of positive associations between residential density, walkability, street connectivity, access to destinations and services, land use mix, pedestrian-friendly features and walking for transport [10]. As a result of its systematic review, the U.S. Community Preventive Services Task Force concluded that several environmental approaches should be utilized to promote PA, including creating or enhancing access to indoor and outdoor resources for PA combined with informational outreach and both street-scale and community-level land use and urban design policies and practices [11,12].

Approaches for examining the associations between the built environment and PA have often focused on a fixed area around where individuals live when identifying environmental exposures; for example, by creating a buffer around a geocoded home address and then characterizing the environmental attributes within the buffer [13]. However, research has shown that people spend 50% of their time away from the home environment and engage in a range of activities in those areas, including PA [14]. Recent studies using GPS and other geospatial technology have examined the potential influence of the built environment in multiple contexts that individuals spend time in throughout the day, including during transportation, work, and leisure [15,16,17]. GPS monitoring enables a spatially and temporally dynamic measurement of activity that mitigates uncertainties associated with static definitions of the geographic context and when integrated with measurements from accelerometer-based devices allows for a more precise identification of where PA occurs [18,19,20].

Recent built environment research using GPS and a dynamic spatial approach varies in design and methods, as standardized methods have not yet been established. One approach is to use individuals’ GPS tracking data to create daily activity spaces, which are defined as the local environments within which individuals circulate during their daily activities. Few studies have specifically used GPS-derived activity spaces to characterize built environment exposures, and the evidence of associations with PA from these studies has been mixed [21,22]. For example, Hirsch and colleagues found statistically significant associations between destinations and step counts when examining activity spaces created around pedestrian and cycling trips among 77 older adults in Vancouver, Canada [23]. However for all modes of travel, the associations were not statistically significant. In another study of 120 adults from Detroit, Zenk and colleagues created two types of activity spaces based on the standard deviational ellipse (SDE) and the daily path area and found no significant associations between the amount of park land within activity spaces and objectively measured moderate or vigorous PA [22]. In another study of adolescents in Vancouver, British Columbia, researchers used a similar daily path area approach by creating a 200 m buffer around GPS trips and found no associations between the size of activity spaces and MVPA assessed with accelerometer-based devices [24].

A key limitation of research on associations between the built environment and PA is that it is predominated by studies that have used a fixed, home-centric spatial approach to characterize the physical environment. Given the lack of widely accepted methods for the combined use of GPS and accelerometer-based devices, the primary aim of this study was to examine associations between the built environment and objective measures of sedentary behavior (SB), light PA (LPA), and MVPA in adults using three approaches for defining daily activity spaces.

## 2. Methods

### 2.1. Design Overview

Participants’ daily activity was assessed with accelerometer-based devices, small GPS units, and travel logs over 7 days. Participants completed a PA and transportation survey at the end of the monitoring period. PA data and built environment exposures within activity spaces were summarized for each monitoring day (i.e., at the person-day level). Associations between the built environment and PA outcomes were analyzed at the person-day level using a repeated measures design.

### 2.2. Participants and Recruitment

The inclusion criteria for participation in this study were as follows: (1) current membership in Boston’s Bluebikes bike share system; (2) 18 years of age and older; and (3) ability to read and write in English. Bluebikes is a public bike share system that was launched in 2011 and operates in the city of Boston and several adjacent towns and cities. Participants were recruited using several methods that included intercepts at bike share stations, fliers posted on the University of Massachusetts Boston campus, a campus-wide email campaign sent to students, and electronic fliers posted online with Boston-based cycling groups. Brief recruitment intercepts were conducted at 16 bike share stations in the Boston neighborhoods of Dorchester, Roxbury, Jamaica Plain, South Boston, South End, and Back Bay. Recruitment was conducted for 3 months from March to June 2019. Out of the sixty-eight individuals who initially expressed interest in the study, 34 provided informed consent and participated in data collection.

### 2.3. Data Collection

Participants were instructed to wear a Columbus V-900 GPS data logger (Victory Technology, Co., Ltd., Guangzhou, China) and an ActiGraph GT9X-BT activity monitor (ActiGraph, Pensacola, FL, USA) for 7 consecutive days. GPS devices were programmed to collect data in 1 s intervals. Participants were instructed to keep the GPS unit on their person during waking hours either in a bag, backpack, or pocket. They were also asked to charge the GPS unit each evening. Participants were asked to wear the ActiGraph device on the hip during all waking hours and to remove it during bathing, sleep, and water activities. Participants received two daily automated text messages to optimize compliance with GPS and activity monitor wear instructions. Participants were also asked to complete a daily travel and wear log to document when the two devices were worn or removed. Equipment, instructions, and log sheets were mailed to participants with a postage-paid return envelope. At the end of the 7-day monitoring period, participants completed an online survey that included questions on transportation, PA, bike share use, self-efficacy for cycling, and demographics. Survey items also assessed specific facilitators for cycling, such as the presence of bike lanes, other bike-specific built environment variables, and perceived safety.

### 2.4. Daily Activity Spaces

**Figure 1:**
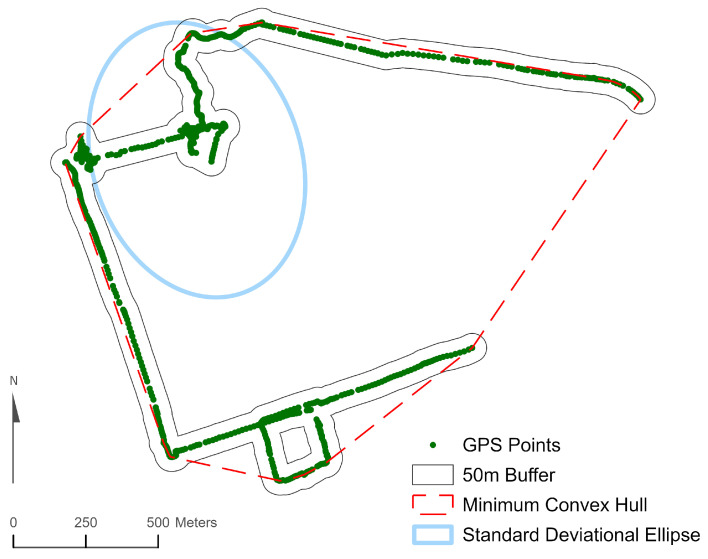
Illustration of 50 m buffered GPS track, minimum convex hull, and standard deviational ellipse activity spaces.

One member of the research team completed visual inspections of the GPS points for each monitoring day and compared these points to participants’ travel log entries. Any instance in which the location indicated by the GPS coordinates did not match the location reported in the travel log was defined as a data discrepancy. Any monitoring days with GPS discrepancies were deemed invalid and excluded from statistical analyses. All GIS data processing was completed using ArcGIS 10.8 (Esri, Redlands, CA, USA). Three GIS approaches were used to create daily activity spaces for participants: (1) a 50 m buffered GPS track; (2) minimum convex hull (MCH), and (3) one standard deviational ellipse (SDE1). Activity spaces reflect the spatial extent of an individual’s activity over time, which could be over a day or week. Although there is no widely accepted standard for creating GPS-based activity spaces, these three approaches are similar to those used in several previous built environment studies using GPS tracking data [22,25,26]. Buffered GPS tracks were generated using the Points-to-Line and Buffer tools in ArcGIS. The MCH method connects the outermost GPS points with a straight line so that there are no concave sides to the resulting polygon The MCH was calculated using the Minimum Bounding Geometry tool in ArcGIS. SDE activity spaces were calculated using the Directional Distribution tool in ArcGIS following the empirical rule whereby SDE1 encompassed approximately 68% of all GPS points on a valid monitoring day. The three types of activity spaces are illustrated in Figure 1.

### 2.5. Built Environment Variables

For each daily activity space, six built environment variables were created using geographic databases from the Massachusetts Department of Transportation, the Massachusetts Bureau of Geographic Information, and Bluebikes. These variables included multi-use trail and bicycle infrastructure densities, intersection density, land use mix, greenness, and bike share docking station density. Two bicycle infrastructure variables were created using bicycle network data for Massachusetts as of the end of 2018 from the MassDOT Open Data Portal: the density of multi-use paths (km of paths per square km within activity spaces) and density of the total bicycle infrastructure, which included shared use paths, bicycle lanes, cycle tracks, and bicycle/pedestrian priority roadways. Counts of intersections with 3 or more connecting road segments within activity spaces were derived from the MassDOT road network layer (MassGIS Data: Massachusetts Department of Transportation (MassDOT) Roads|Mass.gov) after excluding limited-access highways, highway ramps, and tunnels, which are inaccessible to pedestrians and cyclists. The land use mix was calculated using 2016 land cover/land use data from MassGIS. The specific categories of land use/land cover included residential, commercial, industrial, recreational, open space, forest, agricultural, and water. In ArcMap, the identity procedure was used to calculate the total area and proportion for each land use category within the activity spaces. An entropy formula developed by Frank and colleagues was applied to the proportions to create a measure of land-use-mix with values closer to 1.0 indicating greater mix [27].

### 2.6. Physical Activity Outcomes

ActiGraph’s ActiLife software was initially used to convert raw 30 Hz accelerometer-based device data files to 1 sec epoch files. Data were then further processed in R. The 1 sec epoch data were converted to 10 sec and 1 min epochs using the ‘AGread’ R package [28]. Data were then cleaned and examined to remove non-wear time using the Choi algorithm [29,30]. A valid monitoring day was defined as a minimum of 600 min of wear time in a 24 h period. Once the data were cleaned, the 10 sec epochs were used to estimate time spent in sedentary behaviors (<1.5 METs), light PA (LPA; 1.5-2.99 METs), and MVPA (≥3.0 METs), using the ‘TwoRegression’ R package, v1.0.0 [31]. These estimates were made using the 2010 refined Crouter algorithm [32]. Three PA outcomes were derived from the cleaned activity monitoring data. To control for differences in daily monitoring time between participants, we created the following variables: (1) proportion of daily monitoring time that was sedentary, (2) proportion of LPA, and (3) proportion of MVPA.

### 2.7. Covariates

The self-reported socio-demographic covariates included age, gender, race, ethnicity, and family income. Participants reported their height and weight, which were used to calculate body mass index (BMI; kg/m^2^). They also reported whether they perceived their neighborhood to have a high crime rate (yes or no). Three weather variables were examined as potential confounding variables: average daily temperature (Fahrenheit), average daily wind speed (miles per hour), and daily precipitation (inches of rain and snow). Daily weather reports from the Boston, MA weather station (Global Historical Climatology Network Daily Station: USW00014739) were obtained through the National Oceanic and Atmospheric Administration’s National Centers for Environmental Information [33].

### 2.8. Statistical Analyses

All statistical analyses were conducted with SAS software, Version 9.4 (SAS Institute Inc., Cary, NC, USA). The generalized estimating equation (PROC GENMOD) was used to test for associations between built environment variables and the proportion of monitoring time that was sedentary, LPA, and MVPA. Repeated measures on participants at the person-day level were specified using each unique participant ID. Models were created using a compound symmetry covariance structure. A separate model was generated for each built environment variable for the three types of activity spaces. Unadjusted models and final adjusted models that included age, gender, family income, BMI, perceived neighborhood crime, precipitation, and average daily temperature were fit. Multiple testing adjustments were not made, and no confirmatory hypothesis testing was conducted. Associations were considered statistically significant with a *p*-value ≤ 0.05.

## 3. Results

### 3.1. Participant Characteristics

Thirty-three out of 34 initial participants wore the GPS unit and had valid wear days for the accelerometer-based device (≥600 valid wear minutes in a day), which resulted in a total of 168 person-days of monitoring. When the data were then visually screened for inconsistencies between GPS points and daily travel log entries (the operational definition for an invalid monitoring day based on the GPS data), 27 monitoring days, including all days for two participants, were removed from the database. The final analytic database included an accelerometer-based device, GPS, and built environment data for 31 participants who had 141 person-days of monitoring (mean = 4.5 days per person).

Among the 31 participants with valid accelerometer-based device and GPS monitoring days, 19 (61.3%) self-identified as male and 12 (38.7%) as female. About 48% of the participants were between the ages of 20 and 29, with the remaining 52% ranging between 30 and 57 years of age. Twenty-four participants (77.4%) were White, five were Asian, one was Black, and one participant was Biracial (White and Asian). Two participants reported an Hispanic, Latino/a, or Spanish origin. Eleven participants (35.5%) were married, 2 were divorced (6.5%), 2 were members of an unmarried couple (6.5%), and 16 (51.6%) were never married. About 80% of participants (n = 25) were employed full time and 45% (n = 14) had household incomes of $100,000+ per year. Over 90% of the participants had a bachelor’s degree or higher. The mean body mass index (BMI) of participants was 23.8 ± 2.7. Based on the BMI, twenty participants had a “healthy weight”, 10 were “overweight”, and one was “obese”. No participants were current smokers, and only six (19%) were previous smokers. Twelve study participants (38.7%) rated their health as excellent, 15 (48.4%) rated it as very good, 3 (9.7%) rated it as fair, and 1 (3.2%) participant rated their health as poor.

### 3.2. Participants’ Physical Activity

Table 1 shows the PA and sedentary time for weekdays, weekend days, and all days combined. The average daily wear time for participants was 847.1 ± 100.3 min with about 40 more minutes occurring on weekdays compared to weekend days (855.1 ± 100.0 and 814.9 ± 96.5 min, respectively). Over 113 weekdays, participants averaged 147.4 min of daily MVPA, and over 28 weekend days, they averaged 135.7 min of MVPA. The proportion of daily monitoring time that was MVPA was 17.4% on weekdays and 16.5% on weekends. The mean daily proportion of sedentary time during the week was 64.4%, with about a 4% greater proportion of sedentary time occurring during the weekdays than weekends (551.3 min on weekdays versus 488.0 min on weekends). In addition, participants spent 5% more per day engaged in LPA during weekend days than weekdays (191.2 min on weekends versus 156.4 min on weekdays, respectively).

### 3.3. Unadjusted Associations Between the Built Environment and Physical Activity

Table 2 shows the unadjusted associations between the built environment attributes within daily activity spaces and the daily proportion of time participants engaged in SB, LPA, and MVPA. The land use mix within MCH activity spaces had a statistically significant negative association with the proportion of daily time spent being sedentary (*p* = 0.03). No other built environment variable (intersection density, greenness, multi-use trail density, bike infrastructure density, bike station density) was associated with the proportion of SB, regardless of the type of daily activity space.

Greenness within MCH activity spaces had a significant positive association with the proportion of time in LPA (*p* = 0.01). The multi-use trail density within line buffer and MCH activity spaces (*p* = 0.01 and 0.02, respectively) and the bike station density within MCH and SDE activity spaces (*p* = 0.01 and 0.01, respectively) showed significant negative associations with the proportion of LPA. The bike infrastructure density within line buffer activity spaces had a significant negative association with the proportion of LPA (*p* = 0.01). None of the built environment variables (multi-use trail and bicycle infrastructure densities, intersection density, land use mix, greenness, and bike share docking station density) had statistically significant associations with the proportion of the daily monitoring time for MVPA.

### 3.4. Adjusted Associations Between the Built Environment and Physical Activity

Table 3 displays the adjusted associations between the built environment variables and the proportion of time participants engaged in SB, LPA, and MVPA. There were no statistically significant associations (*p* < 0.05) between built environment variables and the proportion of SB in adjusted models. Greenness within MCH activity spaces had a significant positive association with the proportion of monitoring time that was LPA (*p* = 0.02). The multi-use trail density within line buffer (*p* = 0.01) and MCH activity spaces (*p* = 0.05), bike infrastructure density within line buffer activity spaces (*p* = 0.01), and bike station density within MCH activity spaces (*p* = 0.01) showed significant negative associations with the proportion of LPA.

The bike infrastructure density within the SDE activity spaces had a statistically significant positive association with the proportion of daily time engaged in MVPA (*p* = 0.04). No other built environment variable (land use mix, intersection density, greenness, multi-use trail density, bike station density) was associated with the proportion of MVPA time in adjusted analyses, regardless of the type of daily activity space.

After an adjustment for covariates, the positive association between greenness within MCH activity spaces and the proportion of LPA was attenuated along with most other statistically significant associations found in the unadjusted analyses. The only significant association identified exclusively in the adjusted analyses was the positive association between the bike infrastructure density within SDE daily activity spaces and MVPA. For all other variables, a statistical adjustment either slightly attenuated or eliminated the statistically significant associations found in unadjusted analyses.

## 4. Discussion

The aim of this study was to examine associations between built environment variables in three types of GPS-derived daily activity spaces and objective measures of SB, LPA, and MVPA in a sample of adults from Boston. In both unadjusted and adjusted analyses, we observed that greenness was the most consistent positive correlate of the proportion of time engaged in LPA out of the six environmental variables. Several trail and bicycle facility variables were negatively associated with LPA. The only built environment variable that was positively associated with the daily proportion of time in MVPA was the bike infrastructure density, a measure comprised of shared use paths, bicycle lanes, cycle tracks, and bicycle/pedestrian roadways. The only built environment variable associated with the daily proportion of SB was land use mix, which showed a significant negative association in an unadjusted model but was non-significant after a statistical adjustment. The loss of statistical significance and reduced effect size could be attributed to reduced statistical power in multivariable models and the adjustment for potential confounders. For example, younger individuals tended to live in areas with a greater mix of residential, commercial, and other land uses and were likely less sedentary. After adjusting for these confounders, the association between land use mix and the daily proportion of SB may have weakened. A preferred approach to identifying activity spaces from individuals’ GPS data did not clearly emerge in this study. Out of six statistically significant adjusted associations, two were found in line buffer activity spaces, three in MCH spaces, and one in SDE spaces.

Although numerous studies published since 2015 have incorporated GPS and accelerometer-based devices to examine spatially and temporally dynamic associations between the built environment and PA in adults, many of these studies have not necessarily utilized an activity space approach to identify the locations for built environment exposures [20,34,35,36,37,38]. For example, Duncan and colleagues monitored adults in France with accelerometer-based devices and GPS but focused on associations between Walk Scores at GPS-determined trip origins and destinations and the number of accelerometer-derived steps per 10 min [20]. Similarly a study of U.S. women researchers assessed participants with GPS and accelerometer-based devices for 7 days and linked minute-level GPS coordinates to a GIS walkability layer and a NDVI greenness measure [36]. More recently, Boakye and colleagues also used minute-by-minute GPS locational data to characterize built environment exposures and found significant positive associations between variables, such as NDVI and park space, and the objectively measured PA in adults from Washington state [39]. In these three studies, investigators found evidence of positive associations between spatially dynamic measures of the built environment (i.e., variables not based on a fixed location, such as a residential buffer) and PA outcomes—though they used different methods to define the relevant environmental context as compared to the daily activity space approach used in the present study.

We have identified a small number of studies in adults that generated activity spaces from individual GPS monitoring to derive measures of built environment exposures in relation to PA [22,23,26,40,41]. The findings from these previous studies using activity spaces provide the most direct comparison to findings from the current study. In a study with 120 Detroit, Michigan area participants, researchers examined associations between built environment attributes in two types of activity spaces based on SDEs and daily path areas and the mean daily minutes of MVPA [22]. These activity space approaches are comparable to the SDE and buffered GPS track used in the present study. In this prior study, the investigators found no associations between the proportion of municipal park land within two types of activity spaces and MVPA. In addition, no other PA-related built environment variable was examined in this study. In another study of older Canadian adults, Hirsch and colleagues also used daily path areas, which involved creating 200 m buffers around all GPS-derived trips [23]. They examined associations between the density and diversity of destinations within two types of daily path areas, those for trips conducted via all modes of transportation and those from pedestrian and bicycling trips, and accelerometer-derived total PA, daily step counts, and meeting daily recommended step counts. The only statistically significant association that these investigators found was between the diversity of destinations within pedestrian and bicycling activity spaces and daily step counts. Yi and colleagues used a time-weighted kernel density estimation approach to define daily activity spaces for Hispanic women during pregnancy and postpartum and found significant associations between exposure to parks and open space (i.e., greenspace) and objectively measured MVPA [40]. Neither LPA nor SB were included in this study.

Comparable to the present study’s focus on a range of activity intensities, a recently published investigation of adult women from four U.S. sites explored built environment associations with objective measures of SB, LPA, MVPA, and daily steps [26]. The researchers found statistically significant associations between walkability within daily path area activity spaces and both SB and MVPA but no association between walkability and LPA. In this study, the authors reported that the relationships between greenness and LPA and MVPA were both in the positive direction but were not statistically significant. In contrast to these results, we found a statistically significant positive association between greenness in MCH activity spaces and LPA. Greenness was not associated with sedentary behavior in this previous study nor in our study. Another recent investigation by Perchoux and colleagues using activity space methods focused exclusively on SB measured in older Luxembourg adults with accelerometer-based devices [41]. Researchers in this study buffered the road network around participants’ visited locations to define their activity spaces. One of the main findings was a significant negative association between the intersection density within activity spaces and total SB.

Although our findings for land use mix were equivocal from a statistical significance perspective, they generally supported the notion that MCH activity spaces with a greater mix of residential, commercial, and other land uses were negatively associated with the proportion of monitoring time that was sedentary and positively associated with the proportion of time that was LPA and MVPA. Findings from Marquet and colleagues’ recent study appear generally consistent with our data, although they did not explicitly focus on a land use mix variable [26]. These investigators reported mixed evidence of relationships between a walkability measure that included land use diversity as one of its three main components and sedentary time and no evidence of associations between walkability and LPA.

It is challenging to interpret the findings for greenness in the present study; specifically, whereas greenness was positively associated with LPA but it was not related to MVPA. We did not collect information about the specific types of activities contributing to minutes of LPA and MVPA or the locations within activity spaces with greater or lesser greenness—both of which are factors that facilitate a clearer interpretation of the greenness results. A previous study in Massachusetts that monitored participants with accelerometer-based devices and GPS found negative associations between an NDVI measure of greenness in a residential buffer and MVPA accumulated within that buffer [42]. In another analysis of over 700 Boston residents, researchers also found negative associations between greenness in 1 and 2 km residential buffers and self-reported PA [43]. Recent studies have indicated that NDVI measures of greenness, which use an aerial perspective, may not be the optimal approach for examining PA behaviors. For example, several recent studies in China have shown that eye-level measures of greenness, for example, created using Google Street View panoramic streetscape images, are significantly associated with PA outcomes, such as cycling [44,45] and bike share [46]. In contrast to this finding, Lu and colleagues demonstrated that overhead measures of greenness using NDVI, similar to the approach in the present study, were not associated with cycling [45].

At present, there does not appear to be a gold standard approach for the operational definition and measurement of daily activity spaces. For example, the question of whether it is preferable to use GPS monitoring of study participants or another approach that combines self-reported information and GIS techniques seems to depend on the specific research questions and types of PA outcomes being investigated. The current study’s focus on accelerometer-based device measures of PA, irrespective of whether that activity was for recreation or transportation purposes, appears to lend itself to using GPS tracking that captures all or most outdoor movement. However, studies focused on walking or cycling to destinations (i.e., active travel) may not require GPS monitoring. As an example of this approach, Van Heeswijck and colleagues used a combination of self-reported data about the origin, destination and mode of all transportation activities (trips) over a 24 h period and GIS buffering of those trips to identify activity spaces and then examine associations between the built environment and active travel [21]. They found strong negative associations between active transportation and both greenness and land use mix within individual activity spaces. In studies that involve monitoring individuals over numerous days or weeks, it may be less burdensome to have participants carry and charge a small GPS unit compared to having them complete detailed daily travel logs. Newer activity space approaches are also emerging and require further refinement and evaluation. For example, Sila-Nowicka and colleagues recently described the use of a new activity triangle approach to measuring activity spaces [47]. The main thrust of this method is the identification of three significant locations where individuals spend most of their time using GPS data and the formation of an activity triangle that links those locations. The advantages of using this method for studying built environment exposures relevant to PA and SB are not known but likely warrant an assessment in future studies.

There are several strengths and limitations of this study. Two primary strengths include the use of several different GPS-derived activity space approaches to identify built environment exposures and the expanded focus on associations with SB and LPA, in addition to MVPA. As noted, this study’s findings did not clearly point to a preferred type of activity space, which suggests the need to conduct additional measurement studies. The use of outcomes that included SB, LPA, and MVPA is consistent with a growing focus in PA and public health on the 24 Hour Activity Cycle paradigm [48], which explicitly recognizes the interdependencies between these behaviors. Several limitations of this study include the small sample size, the lack of racial/ethnic and socioeconomic diversity in participants, and the fact that participants were a convenience sample of bike share members. Thus, the results may not be generalizable to groups that are more racially or ethnically diverse, have lower incomes or education, other bike share users in greater Boston, or adults who do not use bike share. Additionally, missing GPS data may have biased some of the findings, though the direction of these effects is difficult to ascertain. Several built environment variables, such as land use mix, multi-use trail density, and bike infrastructure density, trended toward significant associations with key outcomes but due to the small sample size had *p* values greater than 0.05. Also, as noted earlier, another limitation was that we did not assess the specific types of activities that contributed to LPA and MVPA. This type of contextual information may have allowed us to better understand certain findings, such as the positive associations between greenness and LPA, as compared to the null associations for greenness and MVPA. Researchers are reaching a point where they can use pattern recognition with data from accelerometer-based devices to detect specific activities—which should facilitate clearer insights into relationships between the built environment and different intensities and types of PA. A final limitation of this study is that none of the three activity space approaches emerged as a clear preferred method to characterize built environment exposures for physical activity.

## 5. Conclusions

This study used a spatially dynamic approach to examine associations between the built environment and the proportion of participants’ daily monitoring time that was SB, LPA, and MVPA. Greenness within MCH activity spaces was significantly associated with LPA, whereas the density of the bike infrastructure within SDE spaces was positively associated with the proportion of daily MVPA. Further research is needed to identify the best practices for using GPS tracking data to create daily activity spaces, including flexible approaches that may depend on the type of PA outcome.

## Figures and Tables

**Table 1:** Physical activity (PA) and sedentary time for adults in Boston, MA based on electronic activity monitoring.

	Total (n = 141 Person-Days)	Weekday (n = 113 Person-Days)	Weekend (n = 28 Person-Days)
Wear time minutes	847.1 ± 100.3	855.1 ± 100.0	814.9 ± 96.5
Proportion of sedentary (%)	63.6 ± 11.6	64.4 ± 11.1	60.3 ± 13.2
Proportion of light intensity (%)	19.2 ± 6.9	18.2 ± 6.4	23.3 ± 7.5
Proportion of moderate-to-vigorous (%)	17.2 ± 7.3	17.4 ± 7.2	16.5 ± 7.9
Sedentary minutes (MET ≤ 1.5)	538.7 ± 117.0	551.3 ± 115.6	488.0 ± 110.4
Light minutes (MET = 1.6–2.9)	163.3 ± 62.9	156.4 ± 59.1	191.2 ± 71.1
Moderate-to-vigorous minutes	145.1 ± 63.1	147.4 ± 61.1	135.7 ± 71.1

**Table 2:** Unadjusted associations between the built environment in three types of daily activity spaces and proportion of time spent in sedentary, light, and moderate-to-vigorous physical activity (n = 141 person days).

Built Environment Variable	Line Buffer		Minimum Convex Hull		Standard Deviational Ellipse	
	Estimate (SE)	*p* Value	Estimate (SE)	*p* Value	Estimate (SE)	*p* Value
Land use mix						
% sedentary	−13.08 (11.65)	0.26	−22.33 (10.22)	0.03	2.56 (9.22)	0.78
% light	1.52 (6.96)	0.83	10.38 (5.74)	0.07	−3.50 (5.41)	0.52
% MVPA	10.54 (5.95)	0.08	10.28 (5.79)	0.08	0.05 (4.74)	0.99
Intersection density						
% sedentary	−0.01 (0.02)	0.55	0.01 (0.03)	0.88	−0.01 (0.02)	0.59
% light	0.01 (0.01)	0.58	−0.02 (0.02)	0.30	−0.01 (0.01)	0.61
% MVPA	0.01 (0.01)	0.50	0.01 (0.02)	0.42	0.01 (0.01)	0.29
Greenness						
% sedentary	−3.20 (17.42)	0.85	−12.01 (12.48)	0.34	0.61 (10.75)	0.95
% light	8.12 (10.56)	0.42	16.48 (5.27)	0.01	4.95 (5.41)	0.36
% MVPA	−4.83 (9.30)	0.60	−4.61 (8.22)	0.57	−5.81 (7.62)	0.45
Multi-use trail density						
% sedentary	0.05 (0.60)	0.93	0.29 (1.30)	0.82	−2.14 (1.68)	0.20
% light	−1.10 (0.34)	0.01	−1.61 (0.67)	0.02	0.56 (0.71)	0.43
% MVPA	1.03 (0.63)	0.10	1.26 (1.23)	0.26	1.67 (1.16)	0.15
Bike infrastructure density						
% sedentary	0.17 (0.29)	0.56	0.05 (0.44)	0.92	−0.52 (0.33)	0.12
% light	−0.28 (0.09)	0.01	−0.27 (0.15)	0.07	−0.01 (0.11)	0.90
% MVPA	0.11 (0.26)	0.68	0.21 (0.35)	0.55	0.50 (0.28)	0.08
Bike station density						
% sedentary	−0.08 (0.28)	0.79	0.18 (0.33)	0.60	0.26 (0.34)	0.45
% light	−0.06 (0.14)	0.69	−0.42 (0.15)	0.01	−0.32 (0.12)	0.01
% MVPA	0.14 (0.18)	0.44	0.23 (0.26)	0.37	0.03 (0.29)	0.91

**Table 3:** Adjusted associations between the built environment in three types of daily activity spaces and proportion of time spent in sedentary, light, and moderate-to-vigorous physical activity (n = 141 person days) *.

Built Environment Variable	Line Buffer		Minimum Convex Hull		Standard Deviational Ellipse	
	Estimate (SE)	*p* Value	Estimate (SE)	*p* Value	Estimate (SE)	*p* Value
Land use mix						
% sedentary	−9.26 (10.62)	0.38	−16.78 (9.33)	0.07	4.54 (8.99)	0.61
% light	0.69 (6.75)	0.92	8.76 (5.19)	0.09	−2.58 (5.78)	0.66
% MVPA	9.21 (5.12)	0.07	9.12 (6.33)	0.15	−1.45 (5.06)	0.77
Intersection density						
% sedentary	−0.01 (0.02)	0.41	−0.004 (0.02)	0.86	−0.01 (0.01)	0.41
% light	0.01 (0.01)	0.58	−0.01 (0.01)	0.34	−0.003 (0.01)	0.68
% MVPA	0.01 (0.01)	0.50	0.02 (0.02)	0.35	0.01 (0.01)	0.14
Greenness						
% sedentary	4.28 (15.08)	0.78	−5.20 (10.01)	0.60	7.31 (8.76)	0.40
% light	2.02 (8.27)	0.81	11.19 (4.85)	0.02	1.48 (4.86)	0.76
% MVPA	−6.24 (9.52)	0.51	−5.78 (7.16)	0.42	−8.51 (6.65)	0.20
Multi-use trail density						
% sedentary	0.12 (0.59)	0.83	−0.33 (1.24)	0.79	−2.37 (1.46)	0.10
% light	−1.05 (0.33)	0.01	−1.19 (0.62)	0.05	0.84 (0.69)	0.22
% MVPA	0.91 (0.59)	0.12	1.50 (1.05)	0.15	1.49 (1.04)	0.15
Bike infrastructure density						
% sedentary	0.12 (0.26)	0.64	−0.05 (0.38)	0.91	−0.56 (0.34)	0.10
% light	−0.25 (0.08)	0.01	−0.25 (0.14)	0.07	0.06 (0.13)	0.66
% MVPA	0.12 (0.22)	0.57	0.29 (0.30)	0.35	0.51 (0.25)	0.04
Bike station density						
% sedentary	−0.13 (0.24)	0.60	0.02 (0.31)	0.94	0.22 (0.36)	0.53
% light	−0.04 (0.12)	0.76	−0.36 (0.14)	0.01	−0.27 (0.14)	0.06
% MVPA	0.16 (0.15)	0.28	0.33 (0.23)	0.16	0.05 (0.26)	0.86

* Adjusted models included age, gender, BMI, family income, perceived safety, precipitation, and ambient temperature.

## Data Availability

The raw data supporting the conclusions of this article may be made available by the authors on request.
